# Author Correction: Physics-informed deep generative learning for quantitative assessment of the retina

**DOI:** 10.1038/s41467-025-56964-x

**Published:** 2025-02-13

**Authors:** Emmeline E. Brown, Andrew A. Guy, Natalie A. Holroyd, Paul W. Sweeney, Lucie Gourmet, Hannah Coleman, Claire Walsh, Athina E. Markaki, Rebecca Shipley, Ranjan Rajendram, Simon Walker-Samuel

**Affiliations:** 1 Centre for Computational Medicine, University College London, London, UK; 2 Moorfields Eye Hospital, London, UK; 3 Department of Engineering, University of Cambridge, Cambridge, UK; 4 Cancer Research UK Cambridge Institute, University of Cambridge, Cambridge, UK; 5 Department of Mechanical Engineering, University College London, London, UK; 6 Institute of Ophthalmology, University College London, London, UK

**Keywords:** Computational models, Machine learning, Software

Correction to: *Nature Communications* 10.1038/s41467-024-50911-y, published online 10 August 2024

In the version of the article initially published, in the Methods “Image dataset” section and in the Acknowledgements, the wrong audit number was reported, when in fact Research on Anonymised Data approval (ROAD17/034) was used in accessing ophthalmological image data from Moorfields Eye Hospital NHS Foundation Trust, as is now amended in the HTML and PDF versions of the article.

