# Supplementary material for: Physics-informed deep generative learning for quantitative assessment of the retina
Source: Nat Commun. 2024 Aug 10;15:6859. doi: 10.1038/s41467-024-50911-y (PMC11316734; doi:10.1038/s41467-024-50911-y)
Supplement: Supplementary file 1 — Supplementary Information [file 41467_2024_50911_MOESM1_ESM.pdf]

Supplementary Information for

Physics-informed deep generative learning for quantitative assessment of the retina

Authors: Emmeline Brown[1,2], Andrew Guy[1,3], Natalie Holroyd[1], Paul W Sweeney[4], Lucie Gourmet[1], Hannah Coleman[1], Claire Walsh[1,5], Athina E Markaki[3], Rebecca Shipley[1,5], Ranjan Rajendram[2,6], Simon Walker-Samuel[1]

[1]Centre for Computational Medicine, University College London, London, UK
[2]Moorfields Eye Hospital, London, UK
[3]Department of Engineering, University of Cambridge, Cambridge, UK
[4]Cancer Research UK Cambridge Institute, University of Cambridge, Cambridge, UK
[5]Department of Mechanical Engineering, University College London, London, UK
[6]Institute of Ophthalmology, University College London, UK

Correspondence: Simon Walker-Samuel (simon.walkersamuel@ucl.ac.uk)

Parameter	Value
Eye geometry	
Optic cup diameter (O_d)	0.7 – 1.2 mm
Optic disc radius	(1.1 – 1.5) O_d
Eye diameter	23 — 25 mm
Optic nerve – fundus displacement	3.5 – 5.5 mm [1]
Fovea radius	500 ± 20 μm
Macula radius	2500 ± 200 μm
Retina radius	30 – 40 mm [2]
L-system	
Central retinal artery diameter	135 ± 15 μm
Central retinal vein diameter	151 ± 15 μm
Central retinal artery initial branching angle	10 ± 5 °
Length of first retinal artery / vein segment	0 — 1500 μm
L-system branching angle	30 ± 2 °
L-system inter-branch distance	(30 ± 3) r_i
Optic disc / fovea angle	6.3 ± 3.0 ° [3]
CCO / LVM	
Murray exponent	2.4 ± 0.1
Macula flow factor	1.5 — 2
Maximum spacing	2500 μm
Minimum spacing	150 μm
Number of refinements	5
Tortuosity: $a_1 \sin(x/p_1) + a_2 \sin(x/p_2)$	
p_1	(15 – 25) r_i
p_2	(30 – 50) r_i
a_1 (artery)	(1.0 – 3.5) r_i
a_1 (vein)	(1.0 – 7.5) r_i
a_2	a_1 * 0.4
Fluid dynamics	
Central retinal artery pressure	60 ± 5 mmHg
Central retinal vein pressure	20 ± 5 mmHg

Supplementary Table 1. Parameters used in our procedural modelling of retinal vasculature informed by metrics from clinical studies [4, 5]. Parameters expressed as ranges were treated as uniform random distributions, whereas values with uncertainties were treated as random normal distributions (mean ± s.d.). r_i is the radius of the ith vessel segment; x is distance along the vessel segment.

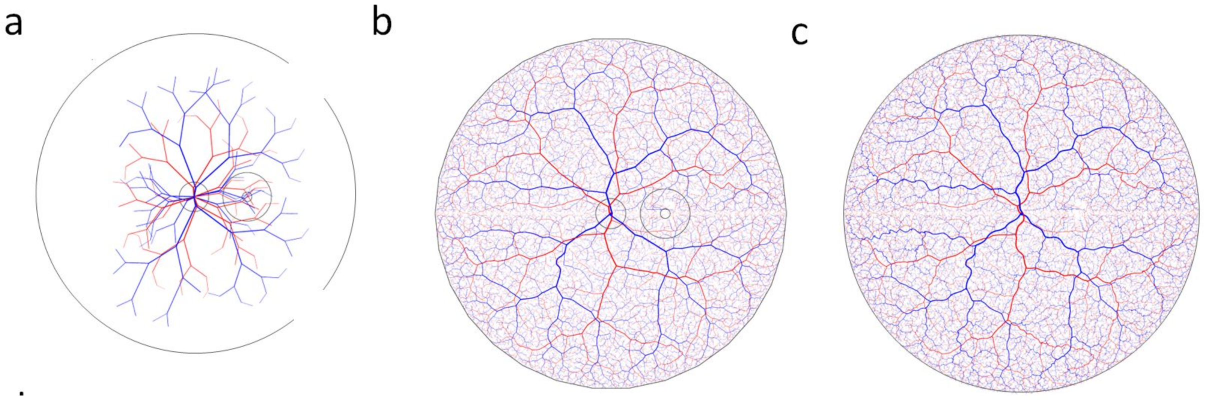

Supplementary Figure 1: **Generation of synthetic retinal vascular networks.** A) An example seeding L-system output, featuring prototype arterial (red) and venous (blue) trees emanating from a central optic disc, and with asymmetric branching towards a macular region offset to the right of the optic disc. B) A result of CCO and LSV algorithms applied to the networks in a). c) Overlay of tortuosity displacements using sinusoidal harmonics on (b).

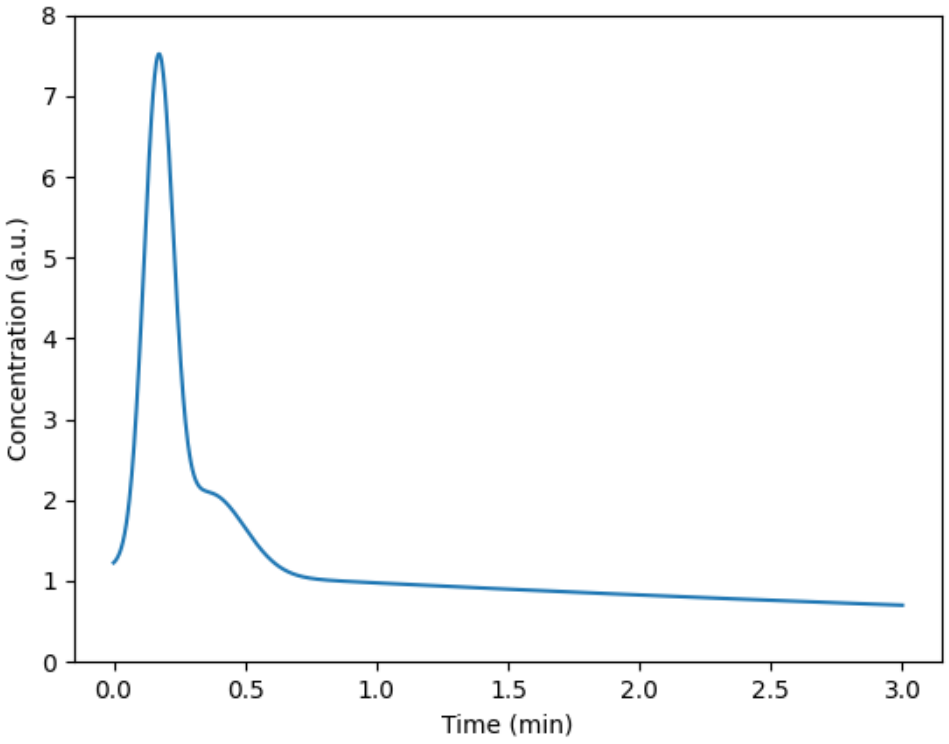

Supplementary Figure 2. Fluorescein arterial input pharmacokinetics used to simulate retinal fluorescein delivery.

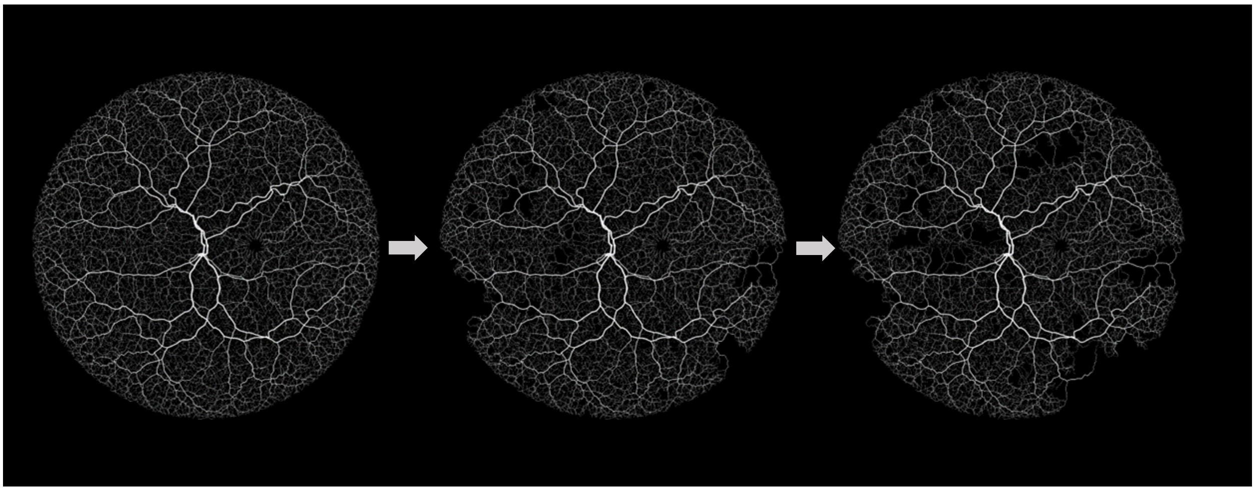

Supplementary Figure 3. Simulated progressive onset of DR in simulated retinal blood vessel networks.

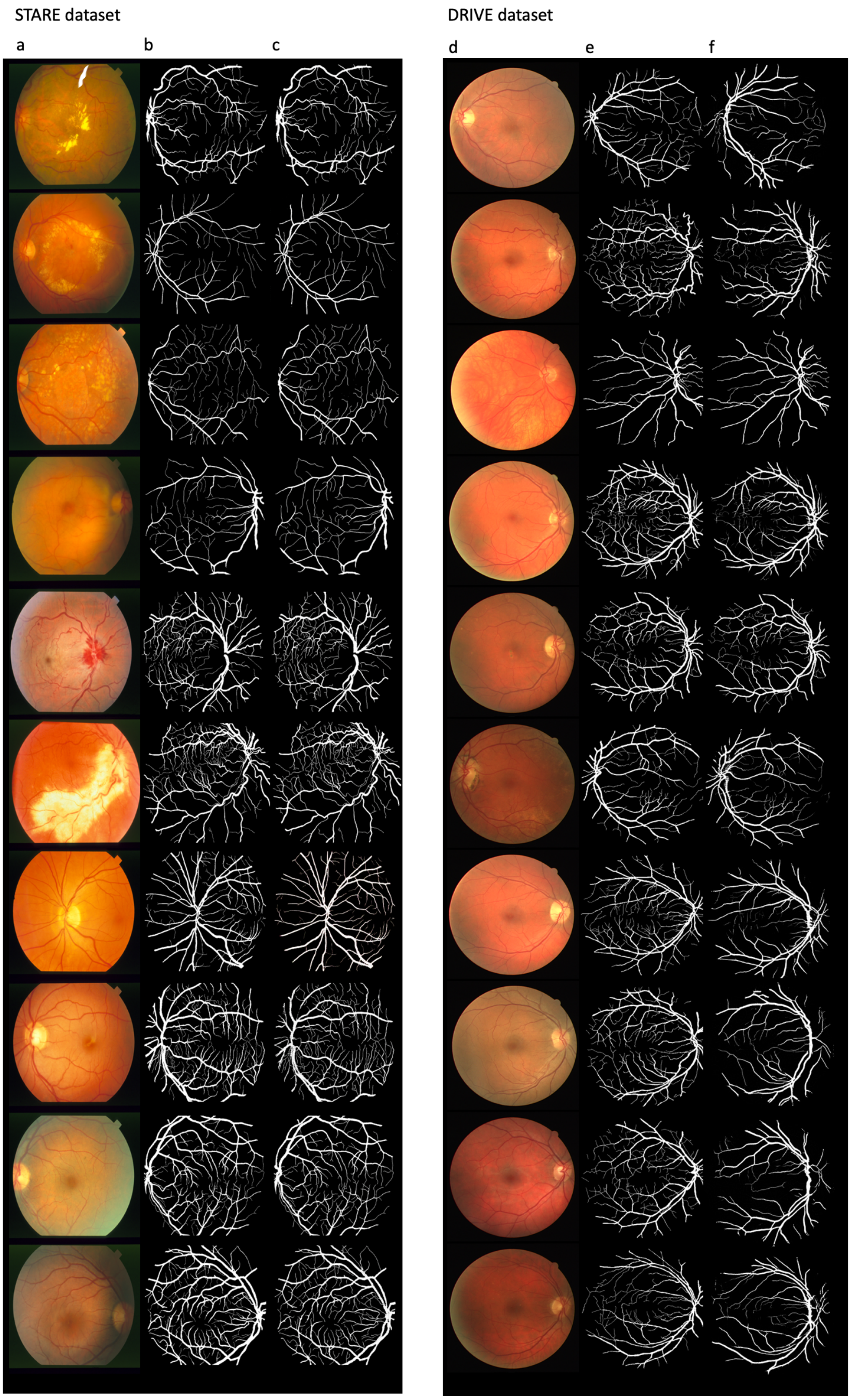

Supplementary Figure 4. Segmentation output of STARE and DRIVE images. a and d) original fundus images from STARE and DRIVE datasets respectively. b and e) manual segmentation image from the public datasets. c and f) PI-GAN based segmentation output.

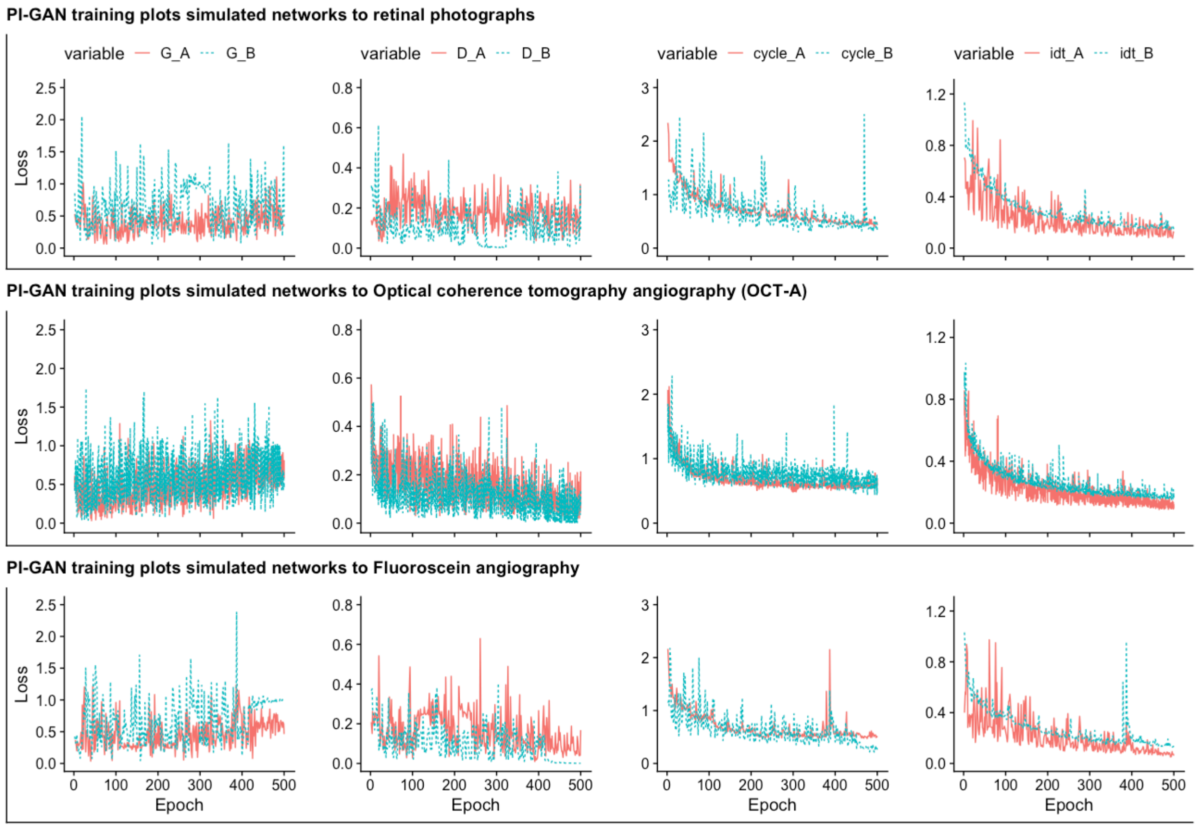

Supplementary Figure 5. **PI-GAN training plots of generator, discriminator, cycle and iteration losses.** Plots show generator losses (G_A, generator loss for conversion of images from domain A to domain B (A→B) and G_B, generator loss for conversion of images from domain B to domain A (B→A)), discriminator losses (D_A, discriminator loss (G_A(A), D_B discriminator loss G_B(B)), cycle consistency (cycle_A (equation lambda_A * ||G_B(G_A(A)) - A||) and cycle_B (equation lambda_B * ||G_A(G_B(B)) - B||) and identity losses defined by equation lambda_identity * (||G_A(B) - B|| * lambda_B + ||G_B(A) - A|| * lambda_A) (idt_A, idt_B). Plots show the losses during training of simulated networks to retinal photographs, OCT-A images, and fluorescein angiography.

	Mean (standard deviation)						Statistical analysis (p values)
	Periphery		Optic disc		Macula		Real v simulated
	Healthy control (n=19)	Simulated (n=100)	Healthy control (n=19)	Simulated (n=100)	Healthy control (n=19)	Simulated (n=100)	ANOVA p value
Branching angle (degrees)	104 (45.30)	114 (40.0)	107 (42.8)	106 (39.4)	106 (39.7)	110 (42.1)	0.824
Inter-branch length (µm)	136 (95.6)	128 (92.3)	143 (146.0)	151 (182.0)	112 (88.5)	125 (75.6)	0.177
Tortuosity	0.485 (0.0437)	0.557 (0.325)	0.488 (0.0517)	0.552 (0.314)	0.488 (0.0345)	0.480 (0.0345)	0.095
Network volume (μm^3)	37058 (48860)	34235 (127146)	36359 (41876)	52145 (182394)	34603 (45212)	34243 (127251)	0.061
Diameter (µm)	100.68 (179.50)	92.11 (150.49)	111.76 (235.23)	101.58 (198.78)	87.43 (296.70)	62.42 (220.18)	0.593

Supplementary Table 2. **Summary statistics (mean, standard deviation) for retinal vessel branching angle, inter-branch length, tortuosity, volume and diameter in three regions of the retina** (periphery, optic disc, macula) and by data type (control, DR, simulation) and ANOVA p values.

Imaging modality	Real versus synthetic FID	Real versus real FID
Retinal photographs	6.95	8.30
OCT-A enface	3.06	4.41
FA	5.17	3.69

Supplementary Table 3: FID scores for real versus synthetic data and real versus real data (used as a baseline). We assigned two groups of real images for each image type using R runif() function. FID was calculated between 1) two sets of retinal photographs, 2) two sets of OCT-A, 3) two sets of FA

Supplementary references

1. Jonas, R.A., et al., *Optic Disc-Fovea Distance, Axial Length and Parapapillary Zones. The Beijing Eye Study 2011.* PLoS One, 2015. **10**(9): p. e0138701.
2. Kolb, H., *Simple Anatomy of the Retina*, in *Webvision: The Organization of the Retina and Visual System*, H. Kolb, E. Fernandez, and R. Nelson, Editors. 1995: Salt Lake City (UT).
3. Choi, J.A., et al., *The foveal position relative to the optic disc and the retinal nerve fiber layer thickness profile in myopia.* Invest Ophthalmol Vis Sci, 2014. **55**(3): p. 1419-26.
4. Miller, D., *Optics and Refraction : a User-Friendly Guide*. 1991, Philadelphia, PA, USA: New York: Gower Medical Pub.
5. Mukherjee, P.K., *Manual of Optics and Refraction.* 2015, New Delhi: Jaypee Brothers Medical Publishers.
