# Supplementary material for: Physics-informed deep generative learning for quantitative assessment of the retina
Source: Nat Commun. 2024 Aug 10;15:6859. doi: 10.1038/s41467-024-50911-y (PMC11316734; doi:10.1038/s41467-024-50911-y)
Supplement: Supplementary file 3 — Description of Additional Supplementary Files [file 41467_2024_50911_MOESM3_ESM.pdf]

Description of Additional Supplementary Files

File Name: Supplementary Movie 1

Description: Video visualisation showing synthetic retinal artery vein vasculature and the simulated pharmacokinetics of fluorescein delivery
