# Supplementary material for: Physics-informed deep generative learning for quantitative assessment of the retina
Source: Nat Commun. 2024 Aug 10;15:6859. doi: 10.1038/s41467-024-50911-y (PMC11316734; doi:10.1038/s41467-024-50911-y)
Supplement: Supplementary file 5 — Reporting Summary [file 41467_2024_50911_MOESM5_ESM.pdf]

nature portfolio

Corresponding author(s): Emmeline Brown/ Simon Walker-Samuel

Last updated by author(s): 23/06/2024

Reporting Summary

Nature Portfolio wishes to improve the reproducibility of the work that we publish. This form provides structure for consistency and transparency in reporting. For further information on Nature Portfolio policies, see our Editorial Policies and the Editorial Policy Checklist.

Statistics

For all statistical analyses, confirm that the following items are present in the figure legend, table legend, main text, or Methods section.

n/a	Confirmed	
[ ]	[x]	The exact sample size (*n*) for each experimental group/condition, given as a discrete number and unit of measurement
[ ]	[x]	A statement on whether measurements were taken from distinct samples or whether the same sample was measured repeatedly
[ ]	[x]	The statistical test(s) used AND whether they are one- or two-sided<br>*Only common tests should be described solely by name; describe more complex techniques in the Methods section.*
[ ]	[x]	A description of all covariates tested
[ ]	[x]	A description of any assumptions or corrections, such as tests of normality and adjustment for multiple comparisons
[ ]	[x]	A full description of the statistical parameters including central tendency (e.g. means) or other basic estimates (e.g. regression coefficient) AND variation (e.g. standard deviation) or associated estimates of uncertainty (e.g. confidence intervals)
[ ]	[x]	For null hypothesis testing, the test statistic (e.g. *F*, *t*, *r*) with confidence intervals, effect sizes, degrees of freedom and *P* value noted<br>*Give P values as exact values whenever suitable.*
[x]	[ ]	For Bayesian analysis, information on the choice of priors and Markov chain Monte Carlo settings
[x]	[ ]	For hierarchical and complex designs, identification of the appropriate level for tests and full reporting of outcomes
[x]	[ ]	Estimates of effect sizes (e.g. Cohen's *d*, Pearson's *r*), indicating how they were calculated

Our web collection on statistics for biologists contains articles on many of the points above.

Software and code

Policy information about availability of computer code

Data collection	Data samples are available in: https://github.com/simonwalkersamuel/retinasim
Data analysis	All code is available in https://github.com/simonwalkersamuel/retinasim/tree/main. RetinaSim, and relies on several libraries: 1) The code in this repository (RetinaSim) is written in python (3.8), and both provides functionality and glues together the other libraries; 2) Reanimate for 1D flow simulation (provided here as a submodule); 3) RetinaGen for procedural modelling of blood vessel networks (provided here as a submodule); 4) Pymira for creating and editing spatial graph structures in python (provided here as a submodule). 5) CycleGAN is provided as a submodule. Installation instruction is provided on the GitHub repository. Deep generative learning was performed using an adaptation of https://github.com/junyanz/pytorch-CycleGAN-and-pix2pix provided in the RetinaSim codebase. A DOI link to this repository has been generated via Zenodo: simonwalkersamuel, Emmeline Brown, & Andrew Guy. (2024). simonwalkersamuel/retinasim: RetinaSim v1.0.0 (v1.0.0). Zenodo. https://doi.org/10.5281/zenodo.12188765

For manuscripts utilizing custom algorithms or software that are central to the research but not yet described in published literature, software must be made available to editors and reviewers. We strongly encourage code deposition in a community repository (e.g. GitHub). See the Nature Portfolio guidelines for submitting code & software for further information.

Data

Policy information about availability of data

All manuscripts must include a data availability statement. This statement should provide the following information, where applicable:

- Accession codes, unique identifiers, or web links for publicly available datasets
- A description of any restrictions on data availability
- For clinical datasets or third party data, please ensure that the statement adheres to our policy

The example retinal simulation data have been deposited on Dropbox https://www.dropbox.com/scl/fo/whwru5rmz8g7cr0h8ytg1/h?rlkey=ynbh2kdhe0pcvpfo6cypm9oc6&dl=0. The Moorfields Eye Hospital data is protected and subject to restrictions of data sharing due to its sensitive nature and is not available for access to the wider research community. DRIVE (https://www.kaggle.com/datasets/andrewmvd/drive-digital-retinal-images-for-vessel-extraction) and STARE (https://cecas.clemson.edu/~ahoover/stare/) datasets are available online.

Research involving human participants, their data, or biological material

Policy information about studies with human participants or human data. See also policy information about sex, gender (identity/presentation), and sexual orientation and race, ethnicity and racism.

Reporting on sex and gender	Findings are applicable to both sexes. Male and female participant data were used without prejudice. Data on sex was recorded and was self-reported. Sex was used as a covariate in statistical analyses, and numbers of males and females included was reported for OCT-A datasets (10 male, 9 female).
Reporting on race, ethnicity, or other socially relevant groupings	No socially relevant categorization variables were used in the study.
Population characteristics	This study was carried out in accordance with the Declaration of Helsinki [71]. Ethical approval of retrospective audit data was obtained through Moorfields Eye Hospital Research and Development Audit number 1078. The audit was authorised by Moorfields Clinical Audit team. Clinical ophthalmological retinal images were obtained from equipment at Moorfields Eye Hospital NHS Trust, London, UK: OCT-A images were obtained from a PLEX Elite 9000 (Carl Zeiss Meditec LLC, Dublin, CA, USA), ultra-wide true color retinal photographs were obtained from Zeiss Clarus 500 Fundus machine (Carl Zeiss Meditec LLC, Dublin, CA, USA), fluorescein angiograms were obtained from Optos widefield camera (Optos, Inc. Marlborough, MA, USA). 19 manually segmented OCT-A images were obtained from healthy controls not ascertained for disease status). These manual segmentations were used in comparison of network structure with simulated networks. Datasets of 570 FA images, 590 colour retinal photographs, 43 OCT-A en-face images, and 130 simulated networks were used in training and testing the PI-GAN algorithm.
Recruitment	Retrospective data was used as part of routine assessment at Moorfields Eye Hospital
Ethics oversight	Moorfields Clinical Audit Team

Note that full information on the approval of the study protocol must also be provided in the manuscript.

Field-specific reporting

Please select the one below that is the best fit for your research. If you are not sure, read the appropriate sections before making your selection.

[x] Life sciences   [ ] Behavioural & social sciences   [ ] Ecological, evolutionary & environmental sciences

For a reference copy of the document with all sections, see nature.com/documents/nr-reporting-summary-flat.pdf

Life sciences study design

All studies must disclose on these points even when the disclosure is negative.

Sample size	19 manually segmented OCT-A images were obtained from healthy controls not ascertained for disease status. These manual segmentations were used in comparison of network structure with simulated networks. The mean age was 39.89 (s.d. 11.25). Datasets of 100 synthetic images, 570 FA images, 590 colour retinal photographs, 43 OCT-A en-face images, and 130 simulated networks were used in training and testing the PI-GAN algorithm. The sample sizes for this study were derived based on datasets used previously in generative deep learning, synthetic data research, and retinal vessel segmentation [Refs 19, 20, 21, 22, 23], for answering the primary and secondary research questions.
Data exclusions	Data were not excluded for any reason.
Replication	Replication was performed using publicly available datasets. PI-GAN was evaluated on retinal photograph data sets STARE and DRIVE with corresponding manual segmentations. These public datasets were acquired with a smaller 45 degree FOV and are widely used in benchmarking vessel segmentation. The code used for generating synthetic data has been made publicly available for reproducibility purposes.
Randomization	Randomization to experimental groups was not performed as it was not appropriate to the study because synthetic and clinical datasets were

	analysed and could not be randomised.
Blinding	Image capture was based on daily practice and did not require blinidng. Annotators for image labelling were blinded to the ground truth and were not involved in image collection. Blinding to synthetic and clinical data status was performed during statistical analysis.

Reporting for specific materials, systems and methods

We require information from authors about some types of materials, experimental systems and methods used in many studies. Here, indicate whether each material, system or method listed is relevant to your study. If you are not sure if a list item applies to your research, read the appropriate section before selecting a response.

Materials & experimental systems

n/a	Involved in the study
☒	☐ Antibodies
☒	☐ Eukaryotic cell lines
☒	☐ Palaeontology and archaeology
☒	☐ Animals and other organisms
☒	☐ Clinical data
☒	☐ Dual use research of concern
☒	☐ Plants

Methods

n/a	Involved in the study
☒	☐ ChIP-seq
☒	☐ Flow cytometry
☒	☐ MRI-based neuroimaging

Plants

Seed stocks	*Report on the source of all seed stocks or other plant material used. If applicable, state the seed stock centre and catalogue number. If plant specimens were collected from the field, describe the collection location, date and sampling procedures.*
Novel plant genotypes	*Describe the methods by which all novel plant genotypes were produced. This includes those generated by transgenic approaches, gene editing, chemical/radiation-based mutagenesis and hybridization. For transgenic lines, describe the transformation method, the number of independent lines analyzed and the generation upon which experiments were performed. For gene-edited lines, describe the editor used, the endogenous sequence targeted for editing, the targeting guide RNA sequence (if applicable) and how the editor was applied.*
Authentication	*Describe any authentication procedures for each seed stock used or novel genotype generated. Describe any experiments used to assess the effect of a mutation and, where applicable, how potential secondary effects (e.g. second site T-DNA insertions, mosiacism, off-target gene editing) were examined.*
